# Reprogenetics, reproductive risks and cultural awareness: what may we learn from Israeli and Croatian medical students?

**DOI:** 10.1186/s12910-019-0427-1

**Published:** 2019-11-27

**Authors:** Miriam Ethel Bentwich, Michal Mashiach-Eizenberg, Ana Borovečki, Frida Simonstein

**Affiliations:** 1 Faculty of Medicine, Bar-Ilan University, Safed Campus, P.O. Box 1589, Safed, Israel; 2 Yezreel Valley Academic College, Afula, Israel; 3 School of Medicine, University of Zagreb, Zagreb, Croatia

**Keywords:** Medical students, Reprogenetics, Cultural awareness, Attitudes

## Abstract

**Background:**

Past studies emphasized the possible cultural influence on attitudes regarding reprogenetics and reproductive risks among medical students who are taken to be “future physicians.” These studies were crafted in order to enhance the knowledge and expand the boundaries of cultural competence. Yet such studies were focused on MS from relatively marginalized cultures, namely either from non-Western developing countries or minority groups in developed countries. The current study sheds light on possible cultural influences of the dominant culture on medical students in two developed countries, potentially with different dominant cultures regarding reprogenetics and reproductive risks: Israel and Croatia.

**Methods:**

Quantitative-statistical analyses were employed, based on anonymous questionnaires completed by 150 first year medical students in Israel and Croatia. The questionnaires pertained to the knowledge and attitudes regarding genetics, reproduction and reproductive risks. These questionnaires were completed before the students were engaged in learning about these topics as part of the curriculum in their medical school.

**Results:**

Substantial differences were revealed between the two groups of medical students. Israeli medical students were less tolerant regarding reproductive risks and more knowledgeable about genetics and reproductive risks than Croatian medical students. For example, while nearly all Israeli medical students (96%) disagreed with the idea that “Screening for reproductive risks in prospective parents is wrong,” less than 40% of their Croatian counterparts shared a similar stance. Similarly, all (100%) Israeli medical students correctly observed that “A carrier of a recessive genetic disease actually has the disease” was wrong, as opposed to only 82% of Croatian students.

**Conclusions:**

By linking applicable theoretical literature to these findings, we suggest that they may reflect the hidden influence of the dominant culture in each country, disguised as part of the “culture of medicine.” Acknowledging and learning about such influence of the dominant culture, may be an important addition to the training of medical students in cultural competence, and specifically their cultural awareness. Such an acknowledgement may also pave the road to drawing the attention of existing physicians regarding a less known yet an important aspect of their cultural competence, insofar as the cultural awareness component is concerned.

## Background

Reprogentics, broadly defined as a scientific field “encompassing genetic technologies that have reproductive implications” and particularly genetic screening tools such as pre-natal genetic diagnosis (PND), entail moral, societal and cultural issues, resulting in variations between and within countries [1]. For instance, a pivotal study of European countries found that social responses toward genetic screening in Europe ranged from acceptance to hostility [2]. Furthermore, researchers have observed that varied attitudes toward genetic diagnosis tests may exist due to cultural influence(s) [3–6]. These studies, therefore, highlight the possible cultural influence underlying the attitudes to genetic diagnosis in general and in the context of reprogenetics, in particular, thereby also emphasizing the importance of *cultural competence* and particularly its emphasis on *cultural awareness* on the part of medical professionals involved in genetic diagnosis and counselling.

Thus, cultural competence is broadly understood as acknowledging and incorporating “the importance of culture, assessment of cross-cultural relations, vigilance toward the dynamics that result from cultural differences, expansion of cultural knowledge, and adaptation of services to meet culturally unique needs” ([7], p., 294).Furthermore, a key component of Campinha-Bacote’s much cited the “process of cultural competence” model, is “cultural awareness,” relating to the ability of the individual health care providers such as physicians and nurses (or MS as future physicians) to acknowledge their *own* cultural underpinnings and not merely the influence of culture on their patients’ perceptions [8]. According to this model, “cultural awareness” is crucial to the overall cultural competence of health care providers since “without being aware of the influence of one’s own cultural or professional values, there is risk that the health care provider may engage in cultural imposition” ([8], p., 182). Indeed, a more recent empirical study, focused on different factors potentially influencing medical students’ clinical decision making, has stressed the importance of improving students’ awareness to their own values in order to help them enhance their clinical decision-making [9]. In fact, overall, training for cultural competence, including its entailed cultural awareness, has been found to be important both at the public health level and the individual-based patient-physician contact and communication level [10–13]. Such training is often taken to constitute an important part of medical ethics teaching for medical students.

There have been a number of studies throughout the past decade that have focused on the attitudes and knowledge of medical students (MS) from varied cultural backgrounds, regarding genetic testing in general, and within the context of reprogenetics in particular [14–18]. These MS, especially at the beginning of their training, find themselves at an important crossroad. On the one hand, they bear the cultural influence of the communities and societies in which they were raised before being immersed in the professional education of medicine. On the other hand, “they may represent the future leaders on medical issues in their communities, and thus their attitudes may influence how entire communities accept genetic testing and genetic research” [14].

Interestingly though, the aforementioned studies have focused more on either ethno-cultural minorities within Western-developed countries (e.g. African-Americans in the US), or non-Western developing regions (e.g. Sub-Saharan Africa, East Asia etc.) [16, 18–20]. As the focus has been on minority groups or on non-Western cultures of developing countries often marginalized in comparison to the dominant Western cultures, these studies concern relatively marginalized cultures. Moreover, with respect to reprogentics and genetic risks, there is a lack of studies that focus on the possible influence of the dominant culture on the attitudes and knowledge of MS located in developed countries with different cultural backgrounds. In fact, even studies concerning medical students’ attitudes towards abortion alone, which pertains to a rather “older” theme than reprogenetics, seldom offer a comparison between two developed countries with different cultural backgrounds [17].

The current study enters a relatively uncharted domain with respect to the issue at stake in developed countries. This study focuses on two such developed countries, which may represent quite different cultural perspectives on genetics, reprogenetics and reproductive risks: Israel and Croatia. Accordingly, in Israel the attitude of the population both in the medical professions and among the general public is largely in favor of PND, and it has also been reported that Israelis are massive consumers of genetic tests, and are leaders of research in novel reprogenetic technology [21–23]. In contrast, in Croatia, there were no studies on genetic testing and especially PND testing on either the general population or among health professionals. Nonetheless, the population in Croatia is considered to be influenced by Catholic church teachings, including the prohibition of abortions since the fetus is considered a whole human being from the moment of conception. For example, a recent poll conducted by the Pew Research Center found that 84% of the population define themselves as Catholics, and that only 40% of the population (and 41% of those who define themselves as Catholics) believe it is either morally acceptable or not a moral issue to perform an abortion [24]. Hence, these preliminary findings suggest that the dominant culture in Croatia, as opposed to Israel, may be less permissive toward reprogenetic technology, and particularly PND, which is designed to give women a choice to perform an abortion in case a genetic disability or a disease is discovered in the fetus.

Specifically, the current study compares the attitudes and knowledge of MS in their first year of study regarding reprogenetics and reproductive risks. The study aims to shed light on differences between medical students in both countries, possibly driven by the dominant culture in each of these developed countries. By highlighting such differences among medical students located in different developed countries, we might attain a better understanding about whether they adopt the views of their dominant culture or whether the position as a MS shapes their views on reprogenetics and genetic risks. If the dominant culture would be found to influence MS, then this influence may be depicted as part of these MS’ *own* culture. Therefore, acknowledging and addressing the possible influence of the dominant culture on MS may be an important addition to the training of MS in cultural competence.

## Methods

### Participants and sampling

The sample included 150 MS, 48 students from Israel (out of 64) and 102 students from Croatia (out of 120), resulting in a response rate of 75 and 85%, respectively. All of the students were at the beginning of their first year of studies. The demographic characteristics of the participants for each group are presented in Table 1. It should be noted that the Israeli medical school participating in the study has a 4-year MD graduate program, whereas the Croatian medical school offers a 6-year undergraduate program. Consequently, it was not surprising to find statistically significant differences between the two groups of students with respect to their age. We further address these differences as well as the relatively small sample that was used in the Results and Discussion sections.

**Table 1:** Comparison of characteristics among the groups

	Israel (*N* = 48)		Croatia (*N* = 102)		
	N	%	N	%	P^a^
Gender					N.S
Male	22	47%	40	39%	
Female	25	53%	62	61%	
Marital status					^b^
Single	38	79%	100	100%	
Married	10	21%	0	0%	
Religion					^b^
Jewish	47	98%	0	0%	
Christian	0	0%	81	79%	
Muslim	1	2%	0	0%	
Buddhist	0	0%	1	1%	
No religion	0	0%	20	20%	
	Mean ± SD		Mean ± SD		
Age (years)	28.6 ± 2.2		20.0 ± 0.8		<.001
min-max	25–36		19–24		

^a^Differences among the groups were tested with Chi-square test for categorical variables and with t-test for continuous variables

^b^The Chi-square *P* value for this variable is not available because of the sample size

### Procedure and instrument

The students were administered a structured self-report questionnaire (see Additional file 1). Originally, the questionnaire was developed for two recently published studies among allied health profession students and laypeople in Israel [23, 25]. Israeli students answered a structured self-report questionnaire in Hebrew, while the Questionnaire for the Croatian students was prepared by a double translation of the questionnaire’s published English version. Both versions of the questionnaire were divided into three sections as described below.

The first part addressed the respondent’s demographic data (see Table 1). The second part targeted the respondent’s attitudes toward genetic syndromes and genetic counseling (Table 2). This part of the questionnaire included 14 statements ranked on a Likert scale between 1 = strongly disagree and 5 = strongly agree. Six of the statements were negative and the others were positive. The overall attitude scores per MS were calculated as the average of all the answers provided by each respondent after the appropriate reversal of the scale for the negative statements. A score close to 1 determined a strong negative position, i.e., strongly against using genetic tools and diagnosis, including the screening of fetuses with genetic disorders, and accordingly a score near 5 expressed the opposite and positive stance toward the use of these tools and diagnosis. The internal consistency (Cronbach’s alpha) of the position score was .79. Finally, the third part targeted the respondent’s understanding of the topic, and included 17 correct/incorrect statements (of which nine were correct and eight were incorrect) (Table 3). Three scores pertaining to the respondent’s understanding were built on the basis of these statements: understanding about a) general genetics (six items), b) reproduction and reproductive tools (five items), and c) reproductive risks (six items). The scores were calculated from the percentage of correct answers provided by the respondents (correct answer = 1; incorrect answer = 0). Previous research reported satisfactory test-retest reliability (0.77, 0.69, and 0.71, respectively, for the three scores pertaining to understanding) [23, 25].

**Table 2:** Attitudes toward genetic tools and reproductive risks among the two study groups

		Disagree	Partly agree	Agree	Missing
1. Screening for reproductive risks in prospective parents is wrong	Israel	95.8	4.2	0	0
	Croatia	38.2	33.3	25.4	2.9
2. It is important to allow parents to select healthy embryos	Israel	4.2	22.9	70.8	2.1
	Croatia	17.6	29.4	53.0	0
3. I would use IVF to select an embryo without breast cancer-related genes	Israel	35.4	20.8	43.8	0
	Croatia	16.7	43.1	39.2	1.0
4. All women planning a pregnancy should test for reproductive risks	Israel	10.4	29.2	60.4	0
	Croatia	20.6	37.3	42.1	0
5. A woman should have prenatal diagnosis if medically indicated (by her age or family history)	Israel	50.0	31.2	18.8	0
	Croatia	2.9	12.7	82.4	2.0
6. Parents should be told results relevant to the health of the fetus	Israel	0	2.1	97.9	0
	Croatia	0	2.9	97.1	0
7. An important goal of genetic counseling is to reduce deleterious genes	Israel	25.0	16.7	56.2	2.1
	Croatia	2.9	22.5	74.6	0
8. It is unfair for a child to be born with a serious genetic disorder	Israel	14.6	14.6	70.8	0
	Croatia	32.3	33.3	32.3	2.0
9. I would continue with the pregnancy if the fetus tested positive for Down’s syndrome	Israel	64.6	25.0	10.4	0
	Croatia	23.5	14.7	59.8	2.0
10. Fetuses with a small defect (such as a missing finger) should be aborted	Israel	89.6	8.3	2.1	0
	Croatia	87.2	6.9	5.9	0
11. I would terminate a pregnancy if the child would be deaf	Israel	66.7	18.7	14.6	0
	Croatia	87.2	9.8	3.0	0
12. Society is improved by the existence of people with disabilities	Israel	35.4	33.3	31.3	0
	Croatia	15.7	46.1	37.3	1.0
13. I would give birth to the child if the fetus were diagnosed with autism (if such a diagnosis was available).	Israel	66.7	22.9	10.4	0
	Croatia	22.6	21.6	55.9	0
14. I would give birth to the child if the fetus were diagnosed with Asperger’s	Israel	45.8	29.2	25.0	0
	Croatia	15.7	19.6	64.7	0

**Table 3:**
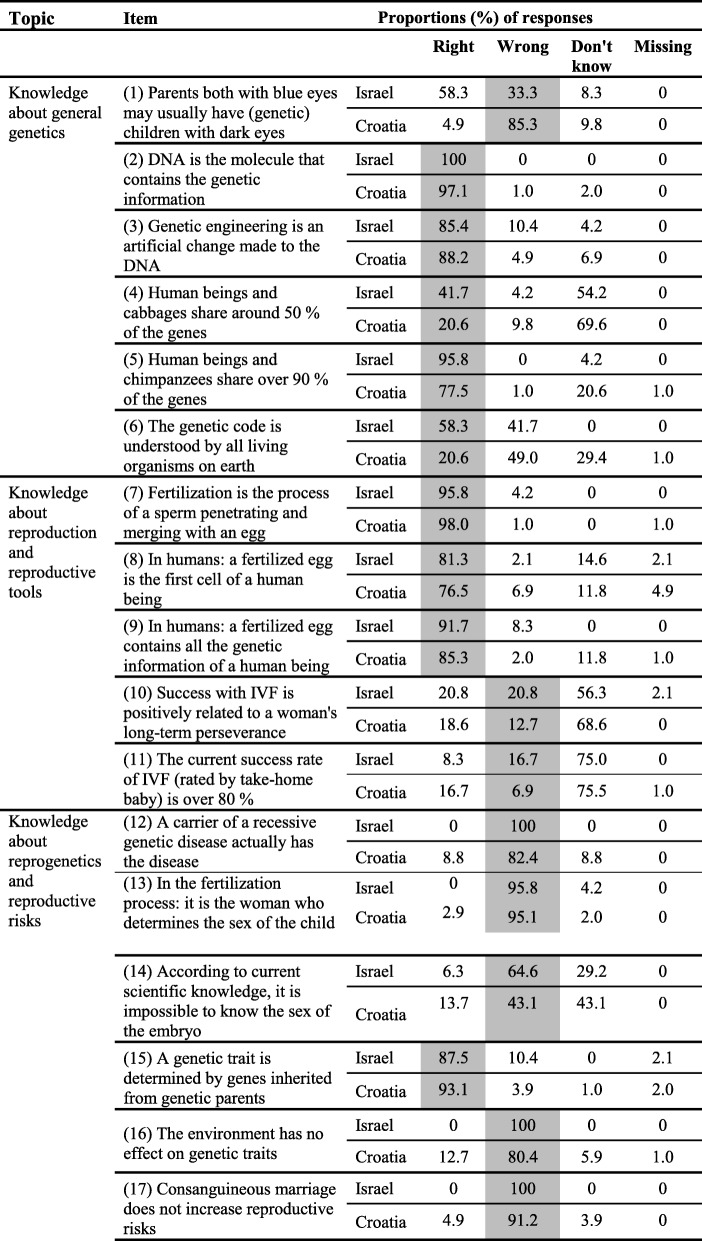
Knowledge about genetics, reproduction, and reproductive risks among the study groups^a^

^a^ The gray background indicates the correct answers

### Data analysis

Analyses were computed using the Predictive Analytics SoftWare (PASW, Version 21.0). Analyses were performed in three steps. First, we explored the distribution of answers for each of the items in the questionnaire regarding attitudes toward genetics, reproduction and reproductive risks and regarding knowledge of genetics, reproduction and reproductive risks. Second, correlation between the attitudes and the knowledge were performed using Pearson correlation. In the third step, group comparisons were performed using ANCOVA’s. Significance was set at the .05 level, and all tests of significance were two-tailed.

### Ethics approval and consent

Ethical approval for the surveys which the students were asked to fill voluntarily and anonymously was granted by the Ethics in Research (human beings) committee of the Faculty of Medicine at Bar-Ilan University (#172015) and by the Central Ethics Committee of the School of Medicine at the University of Zagreb (#386–59–10,106-16-20/267). An implied consent procedure was used, in which students were informed both orally and in a written form about the study, its voluntary nature and that by filling out the questionnaire they consent to participate in the study. More details about the consent procedure that was approved and utilized in this study are provided within the “Ethics Approval and Consent to Participate Declaration” at the end of the manuscript.

## Results

Our results reveal three main themes of interest. First, they show differences in our sample between Israeli and Croatian MS in reference to their attitudes to reprogenetics, and reproductive risks. Israeli students were much less tolerant to reproductive risks, and by extension, more permissive concerning abortions and the use of genetic PND screening, than their Croatian colleagues. Second, insofar as knowledge is concerned, significant differences between these two groups were revealed regarding reproductive risks. Finally, a significant positive correlation (medium effect) between the understanding of reproduction and attitudes to reproductive risks was found only among the Israeli MS.

Thus, regarding the first theme, the attitudes toward reprogenetics and reproductive risks depicted in Table 2 shows that almost all the Israeli MS in the study (96%) disagreed with the idea that “Screening for reproductive risks in prospective parents is wrong.” Hence, nearly all Israeli MS were favorable for such genetic screening. In contrast, less than 40% of the Croatian MS held a similar stance. Similarly, more than two thirds of Israeli MS (71%) believed that parents should be allowed to select healthy embryos and that it would be unfair to the child to be born with a chronic disease or disability, compared to approximately half (53%) and a third (32%), respectively, of the Croatian MS. Likewise, only two thirds (67%) of the Israeli MS rejected this idea to terminate a pregnancy of a deaf child, whereas nearly 90% of the Croatian MS disagreed to terminate such pregnancy. It should also be noted that in order to clearly present the results in this table, we used only three categories of agreement/disagreement representing the original 5-point Likert scale. Hence, the two lowest points on the scale (1–2) were merged into one category: disagree, while the two highest points (4–5) were merged into the “agree” category.

With respect to the differences in knowledge between the two groups of MS, the findings presented in Table 3 show substantial differences between the MS groups concerning more advanced general genetic topics (items 1, and 4–6) and reprogenetics along with reproductive risks (items 12–17). Notice, for example, that 4 out of 6 items in this later domain shows at least 15% difference in favor of the Israeli MS (items 12, 14–16), insofar as their knowledge is concerned. Table 4, which depicts the mean scores for understanding genetics and attitudes toward reproductive risks, further corroborates and demonstrates these findings.

**Table 4:** Means and Standard Deviations for knowledge of genetics and attitudes to reproductive risks

	Israel (*N* = 47)		Croatia (N = 102)		Total (*N* = 149)	
Gender	*M*	*SD*	*M*	*SD*	*M*	*SD*
Knowledge about genetics						
Male (*N* = 62)	72.73	18.22	69.17	18.32	70.43	18.21
Female (*N* = 87)	65.33	14.37	62.22	15.61	63.14	15.23
Total (N = 149)	68.79	16.53	65.00	17.00	66.21	16.89
Knowledge about reproduction						
Male (N = 62)	60.95	18.41	57.30	15.75	58.62	16.70
Female (N = 87)	61.67	19.49	57.19	13.86	58.52	15.74
Total (N = 149)	61.33	18.78	57.23	14.55	58.56	16.09
Knowledge about reproductive risks						
Male (N = 62)	90.48	13.51	87.18	12.37	88.33	12.76
Female (N = 87)	92.67	9.72	78.06	18.28	82.35	17.51
Total (N = 149)	91.67	11.52	81.65	16.75	84.83	15.95
Overall knowledge						
Male (N = 62)	75.29	11.55	71.90	10.25	73.11	10.75
Female (N = 87)	73.77	8.67	67.48	9.49	69.44	9.64
Total (N = 149)	74.47	9.98	69.27	9.98	70.99	10.24
Attitudes toward reproductive risks						
Male (N = 62)	3.57	0.58	2.92	0.51	3.15	0.62
Female (N = 87)	3.47	0.47	2.86	0.58	3.04	0.61
Total (N = 149)	3.52	0.52	2.89	0.55	3.09	0.62

We also performed ANCOVA’s test with groups (Israel and Croatia) as fixed factor, and gender as random factor. Due to age difference between the groups this variable was treated as covariate in the analyses. An initial ANCOVA’s test regarding the attitudes of students revealed a group effect (*F*(1,144) =3.28, *p* = .07, ηp2 = .02), however the *p* value indicated only a statistical tendency, rather than a full statistical significance. We therefore wanted to gain a more accurate understanding of the marginal significance obtained, namely, whether or not it indicates that the age covariate variable may underlie the differences found between the two groups of students (Israeli and Croatian).

In order to achieve this goal, two questions showing essentially no differences in the percentages (Table 2) between the two groups of students were omitted (#6 and #8). This way, the focus of the ANCOVA’s test would be on the remaining 12 questions in which there were supposedly differences between these two groups, so that we would be able to examine whether or not the age covariate variable diminishes the influence of the student group on their attitudes. It is important to note that even though the two questions were omitted, the internal consistency remained the same (Cronbach’s alpha = .79). Indeed, re-performing the ANCOVA’s test showed a primary significant medium effect of group on the average of the attitudes, even when including the age variable as covariate (*F*(1,181) = 18.22, *p* < .001, η_p_^2^ = .09). The Israeli MS in the study had more positive attitudes to genetic screening and use of genetic tools in order to prevent genetic risks (*M* = 3.58, *SD* = 0.64), compared with the Croatian MS (*M* = 2.81, *SD* = 0.52).

We conducted ANCOVA’s test concerning the knowledge variables as well, which included age as covariate and gender as a random factor. A significant main effect of group was also found on the understanding of genetics (*F*(1,137) = 6.94, *p* < .01, η_p_^2^ = .05) and to a lesser extent, on the total score of the knowledge (*F*(1,119) = 4.92, *p* < .05, η_p_^2^ = .04). On average, the Israeli MS knew more than the Croatian MS. Moreover, a significant interaction effect was found on the understanding of genetic risk (*F*(1,140) = 4.50, *p* < .05, η_p_^2^ = .03). On average, among the Israeli MS, women had a better understanding of genetic risk than men, whereas among the Croatian MS, men had a better understanding of genetic risk than women. No significant interaction effect was found for the other variables.

It should be noted that a significant strong effect of gender was found for the average of the attitudes, whether when including all 14 attitudes (*F*(1,4) =17.46, *p* < .05, η_p_^2^ = .81), or when focusing only on the 12 attitudes in which there were differences between the Israeli and Croatian students (*F*(1,3) =42.64, *p* < .01, η_p_^2^ = .92). Hence male students had more positive attitudes to genetic screening and use of genetic tools in order to prevent genetic risks, in both cases (compare relevant rows in Table 4 with *M* = 3.24, *SD* = 0.75 and *M* = 3.10, *SD* = 0.65, for male and female students, respectively). However, unlike the group effect (Croatian vs. Israeli MS), no significant difference was found between the male and female students for the other variables (understanding of genetics, understanding of reproduction, understanding of genetic risk and the total score of the knowledge).

Lastly, with respect to the possible relation between knowledge and attitudes, Table 5 presents correlations between attitudes and knowledge within each of the groups and among the whole sample. The results presented in Table 5 show that the only statistically significant correlation found was among the Israeli MS between the level of understanding of reproduction and attitudes to reproductive risks (r = .3, *p* < .05).

**Table 5:** Pearson correlations between knowledge about genetics, reproduction and reproductive risks and attitudes toward reproductive risks

	Whole Sample (*N* = 150)	Israel (N = 48)	Croatia (N = 102)
Knowledge about genetics	.09	.00	.06
Knowledge about reproduction	.14	.30*	−.03
Knowledge about reproductive risks	.11	.20	−.11
Overall knowledge	.15	.25	−.05

* *p* < .05

## Discussion

In this section, we shall focus on further connecting the differences found between the two groups of MS in relation to the attitudes to and knowledge of reproductive risks with the underlying cultural differences between the dominant cultures of Israel and Croatia. Against this backdrop, we shall suggest and explain why acknowledging and addressing the influence of the dominant culture on future physicians may be important in their medical ethics training, as part of educating them to be genuinely more culturally competent.

As briefly mentioned in the Introduction section, previous studies with the participation of both laypersons and health professionals in Israel have found there is a high tendency to foster genetic tools such as PND and to eliminate reproductive risks, even by means of abortions. In fact, these studies attest to the particular positive attitudes towards genetic screening in the context of reprogenetics among the *majority* of the Jewish population in Israel, already noted in the introduction [21, 22, 26–28]. Consequently, in contemporary Israeli society, with emphasis on the Jewish ethno-cultural majority, genetic testing and screening are generally perceived in a positive light for reducing suffering and increasing the reproductive options of individuals genetically at risk [27, 29]. Accordingly, the Israeli law and its practice is quite permissive with respect to abortions [21]. For instance, while there are professional boards in the hospitals regulating the authorization of abortions, up to the 24th gestational week, abortions can be performed even due to risks for mild defects in the fetus or the physical or psychological wellbeing of the mother [30].

In contrast, in Croatia, even though the law permits abortions, it is substantially more restrictive than the Israeli law. For instance, the upper limit for termination of pregnancy for serious fetal anomaly in Croatia is the 24th gestational week, and abortions are authorized only if congenital anomalies of the fetus will result in a serious mental or physical handicap or if continuing pregnancy will endanger maternal health [31]. In fact, according to one poll conducted by a leading newspaper in the country, it was estimated that two thirds of the doctors in Zagreb, the capital of Croatia, and as many as 95% of their colleagues in Croatia’s second-largest city, Split, refuse to carry out abortions, citing their right to do so on ethical, religious or moral grounds [32]. Furthermore, in a study on genetic engineering with the participation of 493 students, including medical students of different faculties at Zagreb University, found that the majority of the students from all faculties were critical about the use of genetic engineering in general [33].

The findings of such differences between the MS of these countries in our study are further supported by our results which show a statistically significant positive correlation between knowledge and attitudes to reproductive risks only among Israeli MS. Hence, being aware of reproductive risks is not sufficient in itself in order to elicit a positive attitude toward genetic tools and diagnosis on the basis of which unwanted reproductive risks found in the fetus may be eliminated through abortion. Namely the cultural environment might play a role, where in the Israeli-Jewish case the attitude is positive toward such tools and diagnosis, and in the Croatian case it is more reluctant.

Moreover, delving into the content of the only two questions regarding attitudes, for which essentially no differences were found between the two groups of students (according to Table 2), may further emphasize the differences that were found in the other questions. Hence, it could be argued that these questions reflect common perspectives to both groups of students precisely because they less involve the potential cultural gap between the two societies. Instead, these questions pertain to two core values in contemporary medical ethics: autonomy and non-maleficence [34]. Both of these values transcend specific cultural underpinning and are indeed acknowledged in both countries (Israel and Croatia) [35–37]. Thus, the first question pertaining to the statement “parents should be told results relevant to the health of the fetus,” might have been understood by the students in terms of respecting the general parents’ personal autonomy, namely the parents’ right to make their own choice(s). A key part in respecting autonomy in the context of healthcare is the commitment to inform the patients so that they would be able to make informed decisions and thereby exercise their autonomy. The other question referred to the statement “fetuses with a small defect (such as a missing finger) should be aborted,” with which an overwhelming majority of MS in both countries disagreed. This statement’s emphasis is on a “small defect” alone, may have triggered the general “do no harm” directive or the principle of “non-maleficence.” According to this directive or principle, first of all, physicians are committed to causing no harm to their patients. Therefore, in the face of “aborting” the fetus life for a genetic problem that constitutes merely a “small defect,” the commitment to “do no harm” may have been implicitly emphasized from the students’ perspective.

Taken together, the described results correspond with and further corroborate the idea that professional cultures are variations of the dominant culture focused on specific sectors of society and social problems [38, 39]. This is because the mainstream (or dominant) cultural norms and values are instilled within the training frameworks of professionals. Thus, claims have been made that the professional culture of medicine is often perceived by medical professionals as a “culture of no culture,” which may be deemed, particularly by MS, as a “safe zone” from unwanted and feared “cultural complexities” [38, 40, 41]. Consequently, when the dominant culture’s perception of reprogenetics and reproductive risks is disguised under the culture of medicine and understood as “culture of no culture,” it is harder and perhaps even impossible for MS to acknowledge that such perception is actually a culturally-driven viewpoint, at least to a certain extent.

Since MS in both countries align themselves with the dominant culture’s viewpoint on reprogenetics and reproductive risks, our study’s results highlight the possible influence of the dominant culture as a new important consideration when advocating for cultural awareness as part of the overall cultural competence among MS. As we may recall, an important aspect of cultural awareness according to Campinha-Bocte’s model about cultural competence is the ability of the individual physicians (or MS as future physicians) to acknowledge their *own* cultural underpinnings and not merely the influence of culture on their patients’ perceptions [8].

Moreover, our results suggest that by aligning themselves with the dominant culture’s viewpoint in each country, MS may face a substantial barrier to the required awareness of the cultural underpinning of their own perceptions regarding reprogenetics and reproductive risks. In fact, this sort of barrier, driven by MS alignment with the dominant culture’s viewpoint, corresponds with one facet of the tensions described in Schwartz’s “Theory of Cultural Value Orientations.”

According to this theory, three main problems which any cross-cultural society faces can be handled by bipolar value-based cultural perspectives, thereby creating value-based tensions in handling these problems [42]. One such tension concerns the issue of regulating how people manage their relations to the natural and social world, whereby one end of the cultural spectrum asserts the value of “Harmony,” whereas the other end asserts the value of “Mastery.” According to Schwartz, the latter culturally driven value is defined as the “active self-assertion in order to master, direct, and change the natural and social environment to attain group or personal goals” ([42], p., 141) Hence this value can be understood as echoing a situation where the dominant culture’s perspective is the sole viewpoint that counts, which precisely occurs when MS are aligned only with this perspective. Indeed, “harmony,” the contrasting cultural value, seems to be related to a cultural perspective that fosters *cultural awareness* that is part of fostering *cultural competence*, since the value of harmony is described as “fitting into the world as it is, trying to *understand and appreciate rather than to change, direct*, or to exploit” ([42], p.141(emphasis added)).

Therefore, increasing the awareness of MS to the possible influence of the dominant culture on their own cultural underpinnings may be understood as an important part of enhancing their cultural competence. At the same time, training for cultural competence, including cultural awareness, has been found to be important both at the public health level and the individual-based patient-physician contact and communication level [10–13]. Therefore, an increase in the awareness of MS to the possible influence of the dominant culture on their own cultural underpinning may also be understood as part of improving their overall cultural competence, aimed at enhancing their communication with patients. In a similar vein, increasing the awareness of current physicians (and not merely MS as future physicians) to the possible influence of the dominant culture on their own cultural underpinning may be an important extension to their knowledge of and awareness to cultural competence.

It may be suggested, therefore, that the results of our study may potentially point to the need to specifically highlight the possible disguise of the dominant culture as the culture of medicine with respect to reproductive risks and reprogenetics. This should be implemented as part of extending the knowledge of and awareness to cultural competence among future and existing physicians in a given country, whether it be Israel, Croatia or any other state, whose dominant culture advocates a particular perception of reprogenetics and reproductive risks. Only when Israeli future physicians (MS) and current physicians will be made aware of the particular cultural underpinnings of the dominant Israeli-Jewish culture, which so enthusiastically fosters genetic tools and diagnosis, will they become culturally competent regarding such an important and fundamental issue as reprogenetics and reproductive risks. In a similar vein, it may be the case that, only when Croatian future physicians (MS) and existing physicians will learn about the cultural influences possibly underlying their reluctance to use genetic tools and diagnosis in the context of reprogenetics, will they be able to demonstrate a genuine cultural competence in such sensitive and significant issues.

### Study limitations

One possible limitation of this study is its potential lack of representability, as the sample was obtained from only one medical school in each country. In addition, although the questionnaire was tested in previous research on the population in Israel [23, 25], the sample size is rather small and does not allow to examine whether the structure of the measurment tool is similar in the Croatian population. Nonetheless, with reference to the Israeli participants in the research, similarities were found between attitudes of Israeli MS in the present study and a previous similar study focusing on Israeli students of Allied Healthcare Professions, as well as a study on attitudes toward genetic tools among the Israeli population [23, 25]. This suggests a common cultural and societal basis, thereby supporting the representability of our findings, at least for the Israeli part of the study. In addition, insofar as the Croatian part of the study is concerned, and as noted above, previous studies and surveys have shown a tendency among the Croatian population to be more conservative regarding abortions and genetic engineering. Such results fit with our findings regarding the more conservative stance of Croatian MS regarding genetic screening in the context of reprogenetics, thereby strengthening our findings as well. Future studies may also wish to focus on either graduate or undergraduate programs separately in order to get a more accurate depiction regarding advanced knowledge of genetics. Given this limitation in our study, we did not ascribe importance to the results regarding the gaps in advanced general genetics knowledge between the Israeli and the Croatian MS. Still, we believe the findings of this paper illuminate an important and less accounted for aspect of the potential influence the dominant culture may have on future physicians with respect to their perceptions of reprogeentics and reproductive risks.

## Conclusions

Our results show there may be key differences between Israeli and Croatian MS regarding their attitudes to reprogenetics and reproductive risks and their knowledge of reproductive risks. By linking these results to applicable theoretical literature, we suggest that they demonstrate a new important aspect in cultural competence that should be echoed in the training of MS as future physicians, and in the knowledge in cultural competence being made available to existing physicians. We further claim that only by highlighting the cultural underpinnings of the dominant culture regarding themes like reprogenetics and reproductive risks in any given country, and distinguishing them from what is perceived to be the “culture of medicine,” will future and current physicians be able to acquire genuine cultural competence regarding sensitive themes such as reprogenetics and reproductive risks.

## Supplementary information

**Additional file 1.** Questionnaire used in the study.

## Data Availability

The datasets generated or analyzed during this study are not publically available since they use foreign language. However, the datasets are available from the corresponding author on reasonable request.

## Abbreviation

MS: medical students
