# Supplementary material for: Reprogenetics, reproductive risks and cultural awareness: what may we learn from Israeli and Croatian medical students?
Source: BMC Med Ethics. 2019 Nov 27;20:85. doi: 10.1186/s12910-019-0427-1 (PMC6880344; doi:10.1186/s12910-019-0427-1)
Supplement: Supplementary file 1 — Additional file 1. Questionnaire used in the study. [file 12910_2019_427_MOESM1_ESM.docx]

**Part A:**

Gender:

1. Male
2. Female

Birth year:

Marital status:

1. Single
2. Married / live with a partner
3. Divorced /Separated
4. Widowed

Number of children:

Level of religiosity:

1. Very Religious
2. Religious
3. Traditional
4. Secular

Religion

1. Not subscribed to any religion
2. Christian
3. Muslim
4. Druze
5. Buddhist
6. Jewish
7. Other (explain)_______________

Education:

1. Non-academic
2. Academic

Is there a child with special needs in your extended family?

1. No
2. Yes

If yes explain_____________________________________

**Part B.**

This part includes statements which relate to genetics and reproduction. Mark with a circumference your preferred rate for each of the statements. Note that in this part of the questionnaire there is no right or wrong answers. Only your preferred choice is valid.

|  | 1  Strongly disagree | 2  Disagree | 3  Partly agree | 4  Agree | 5  Strongly agree |
| --- | --- | --- | --- | --- | --- |
| 1. Screening for reproductive risks in prospective parents is wrong |  |  |  |  |  |
| 1. It is important to allow parents to select healthy embryos |  |  |  |  |  |
| 1. I would use IVF to select an embryo without breast cancer-related genes |  |  |  |  |  |
| 1. All women planning a pregnancy should test for reproductive risks |  |  |  |  |  |
| 1. A woman should have prenatal diagnosis if medically indicated (by her age or family history) |  |  |  |  |  |
| 1. Parents should be told results relevant to the health of the fetus |  |  |  |  |  |
| 1. An important goal of genetic counseling is to reduce deleterious genes |  |  |  |  |  |
| 1. It is unfair for a child to be born with a serious genetic disorder |  |  |  |  |  |
| 1. I would continue with the pregnancy if the fetus tested positive for Down’s syndrome |  |  |  |  |  |
| 1. Fetuses with a small defect (such as a missing finger) should be aborted |  |  |  |  |  |
| 1. I would terminate a pregnancy if the child would be deaf |  |  |  |  |  |
| 1. Society is improved by the existence of people with disabilities |  |  |  |  |  |
| 1. I would give birth to the child if the fetus were diagnosed with autism (if such a diagnosis was available). |  |  |  |  |  |
| 1. I would give birth to the child if the fetus were diagnosed with Asperger's |  |  |  |  |  |

**Part C**

This part presents statements related to Genetics and reproduction. Mark with an X in the appropriate box, whether you think the phrase is right or wrong. If you do not know the answer please mark the ‘don’t know’ box.

|  | Right | Wrong | Don't know |
| --- | --- | --- | --- |
| 1. Parents both with blue eyes may usually have (genetic) children with dark eyes |  |  |  |
| 2. A carrier of a recessive genetic disease actually has the disease |  |  |  |
| 3. Fertilization is the process of a sperm penetrating and merging with an egg |  |  |  |
| 4. In the fertilization process: it is the woman who determines the sex of the child |  |  |  |
| 5. In humans: a fertilized egg is the first cell of a human being |  |  |  |
| 6. In humans: a fertilized egg contains all the genetic information of a human being |  |  |  |
| 7. According to current scientific knowledge, it is impossible to know the sex of the embryo |  |  |  |
| 8. DNA is the molecule that contains the genetic information |  |  |  |
| 9. Genetic engineering is an artificial change made to the DNA |  |  |  |
| 10. Human beings and cabbages share around 50 % of genes |  |  |  |
| 11. Success with IVF is positively related to a woman's long-term perseverance |  |  |  |
| 12. Human beings and chimpanzees share over 90 % of genes |  |  |  |
| 13. A genetic trait is determined by genes inherited from genetic parents |  |  |  |
| 14. The environment has no effect on genetic traits |  |  |  |
| 15. The genetic code is understood by all living organisms on earth |  |  |  |
| 16. The current success rate of IVF (rated by take-home baby) is over 80 % |  |  |  |
| 17. Consanguineous marriage does not increase genetic risk |  |  |  |
